# Prevalence of Glucose-6-Phosphate Dehydrogenase Deficiency Among Children in Eastern Saudi Arabia

**DOI:** 10.7759/cureus.11235

**Published:** 2020-10-29

**Authors:** Muneer H Albagshi, Suad Alomran, Somaya Sloma, Murtadha Albagshi, Abdullah Alsuweel, Helal AlKhalaf

**Affiliations:** 1 Department of Pediatric Hematology, Hereditary Blood Diseases Center, Al-Ahsa, SAU; 2 Department of Laboratories and Blood Bank, Maternity and Children Hospital, Al-Ahsa, SAU; 3 Department of Pediatrics, Maternity and Children Hospital, Al-Ahsa, SAU; 4 Department of Hematologic Technology, Maternity and Children Hospital, Al-Ahsa, SAU

**Keywords:** glucose-6-phosphate dehydrogenase (g6pd), hemolysis, saudi arabia, children, prevalence

## Abstract

Background

Glucose-6-phosphate dehydrogenase (G6PD) deficiency is the most common red blood cell enzyme deficiency worldwide. The disease is widely distributed in regions where malaria is prevalent, affecting mostly males because the enzyme is inherited as an X-link recessive pattern. In Saudi Arabia, we lack newborn screening (NBS) for G6PD deficiency, despite early reports about high prevalence.

Methods

This is a 10-year retrospective study of children who were screened for G6PD deficiency during their hospitalization between January 2008 to December 2017. The test was carried out using a qualitative fluorescence test suitable for mass screening to determine the prevalence of G6PD deficiency among the admitted children between 0 and 14 years of age.

Results

A total of 48,889 patients were screened which included 27,634 (56.5%) males and 21,255 (43.5%) females with a mean age of 1.93 + 3.98 years. The overall prevalence of G6PD deficiency was 25%, whereas it was 33.8% in the male subset and 13.2% in the female subset. Male sex was significantly correlated with G6PD deficiency. A total of 25,628 newborns were screened, with 14,219 (55.5%) males and 11,409 (44.5%) females, who had a G6PD deficiency prevalence of 18.8%. There was a G6PD deficiency prevalence of 26% in males and 9.9% in females.

Conclusion

The present study confirms the high prevalence of G6PD deficiency in our community. Therefore, we need to establish an NBS program to screen for G6PD deficiency in order to prevent neonatal hyperbilirubinemia encephalopathy, avoidable hemolytic episodes, and to increase awareness among health practitioners.

## Introduction

**Figure 1:**
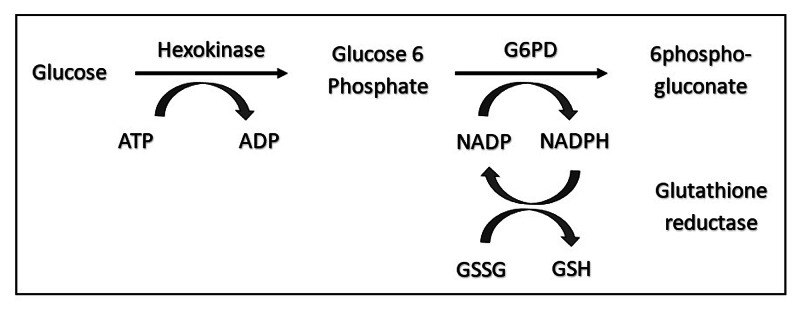
The glucose-6-phosphate dehydrogenase (G6PD) enzyme is the main regulator in the pentose phosphate pathway to maintain NADPH production which helps protect the red blood cells against oxidative damage 6-PG: 6-phosphogluconate; ADP: adenosine diphosphate; ATP: adenosine triphosphate; G-6-P: glucose-6-phosphate; GSH: glutathione; GSSG: glutathione disulfide; NADP: nicotinamide adenine dinucleotide phosphate; NADPH: nicotinamide adenine dinucleotide phosphate

The enzyme glucose-6-phosphate dehydrogenase (G6PD) was discovered in 1932 by Walter Christian and Otto Warburg in red blood cells and yeast [1]. Moreover, severe anemia and hemoglobinuria were observed in children who ingested fava beans by pediatricians in Greece, Portugal, and Italy [2-3]. Favism is the term used to describe hemolysis triggered by the ingestion of fava beans in G6PD deficient individuals, and it ordinarily runs in families. Sulfa drugs used in treatment, as well as prophylaxis for malaria, lead to severe hemolysis [1]. The relationship between primaquine-sensitive patients and the G6PD enzyme was found to be < 15% of normal activity [4]. The G6PD gene is located on X.28 and inherited as an X-linked recessive [5]. G6PD is a housekeeping enzyme found in all human cells. Nicotinamide adenine dinucleotide phosphate (NADP) is reduced to NADPH in the pentose phosphate pathway. NADPH protects the red blood cells from oxidative damage [6-8]. This is the only pathway to generate NADPH. Therefore, in patients who are deficient in G6PD, these cells are damaged and undergo lysis, leading to acute hemolysis (Figure 1). In patients with G6PD deficiency with malaria, G6PD status does not impact baseline hemoglobin, parasitemia, temperature, or the outcomes of antimalarial therapy. The worldwide distribution of G6PD deficiency is mostly related to genetic abnormalities, ethnicity, and population migration. The relationship with malarial areas is obvious in Africa, South Asia, and the Middle East [9-10]. The prevalence of G6PD deficiency is variable among the studied population, from less than 1% in Japan to up to 70% in Kurdish Jews [11-13]. In Saudi Arabia, the prevalence was 4.76% in a cross-sectional study and 14.7% - 30.6% among newborns screened in Al-Ahsa and Qatif over one year [14-16]. In this study, we will review the prevalence of G6PD deficiency among all patients admitted to the Maternity and Children Hospital.

## Materials and methods

We reviewed all G6PD test results that were performed for the pediatric patients who attended the Maternity and Children Hospital in Al-Ahsa, Saudi Arabia, which is the main hospital for children and women's health. This retrospective study covered a 10-year period from January 2008 to December 2017. The pediatric population was defined as the age between 0 and 14 years.

We used the qualitative G6PD screening method by a fluorescence test suitable for mass screening in areas with a high prevalence of G6PD deficiency [17-18].

It used 5 microliters of whole blood with ethylenediaminetetraacetic acid (EDTA) anticoagulant added and mixed with 100 microliters of reagent at room temperature (25ºC) for 10 minutes. Ten microliters of the resulting solution were then placed on the paper provided, which was left to dry. After about 15 minutes in an incubator at 37ºC with ultralight, the sample would fluoresce if G6PD was normal and would not fluoresce if G6PD was deficient. The reagent kits used were Cat Ref. #038F from United Diagnostic Industries, Dammam, Saudi Arabia.

Statistical methods

The descriptive statistics and tables were created using Microsoft Excel MSO 2016 (16.0.4.266.1001) (Microsoft® Corp., Redmond, WA). Data are expressed as mean + standard deviation (SD). The comparison between G6PD deficient and non-deficient patients was made using the Student's t-test for parametric data and the Mann-Whitney U test for nonparametric data. A descriptive analysis of the percentages of categorical variables was done. A p-value < 0.005 was considered to be statistically significant in all comparisons and was calculated using Epi Info™ software, version 7.2.2.7 from the Center for Disease Control and Prevention (CDC). Linear regression analysis was used for correlations.

## Results

This was a retrospective study that was conducted at the Maternity and Children Hospital of Al-Ahsa, Saudi Arabia, which is the major hospital caring for children up to 14 years of age and women. There was a total of 58,431 patients admitted to the hospital over the 10-year period of the study who had been screened for G6PD deficiency. There were 209 samples with incomplete data, and these samples were excluded from the study. Furthermore, we excluded children older than 14 years of age. A total of 48,889 samples were fully analyzed, which included 27,634 (56.5%) male and 21,255 (43.5%) female subjects with a mean age of 1.93 + 3.98 years. The overall prevalence of G6PD deficiency was 25%, with a prevalence of 33.8% among males and 13.5% among females (p < 0.0001) (Table 1). A total of 25,628 newborn patients were admitted to the intensive care or intermediate care units, with males accounting for 55.5% and females accounting for 44.5% with an overall prevalence of G6PD deficiency of 18.8%. Males had a higher rate of G6PD deficiency (26%) compared to females (9.9%) (p < 0.0001) (Table 2).

**Table 1:** Prevalence of Glucose-6-Phosphate Dehydrogenase (G6PD) Deficiency in all Pediatric Patients

Gender	G6PD status		Total
	Deficient	Normal	
Male	9,340 (33.8%)	18,294 (66.2%)	27,634 (100%)
Female	2,864 (13.5%)	18,391 (86.5%)	21,255 (100%)
Total	12,204 (25%)	36,685 (75%)	48,889 (100%)

**Table 2:** Prevalence of Glucose-6-Phosphate Dehydrogenase (G6PD) Deficiency in Newborn Patients

Gender	G6PD status		Total
	Deficient	Normal	
Male	3,687 (26%)	10,532 (74%)	14,219 (100%)
Female	1,133 (9.9%)	10,276 (90.1%)	11,409 (100%)
Total	4,820 (18.8%)	20,808 (81.2%)	25,628 (100%)

## Discussion

G6PD deficiency is the most common red blood cell enzyme deficiency worldwide, affecting over 400 million individuals across the globe [19]. The clinical expression of G6PD deficiency encompasses a spectrum of disease severity related to the ability of red cells to generate NADPH (Figure 1) [3]. Likewise, the observed prevalence of 25% among the pediatric population is similar to previously reported studies in the Eastern province [15, 20], which is conversely higher than that reported in the Central, Northern, and Western regions of Saudi Arabia [20-21]. At the same time, the female pediatric population has a higher rate of G6PD deficiency (13.5%) than the rest of the country, which can be explained by the selective nature of the study, as well as the higher rate of consanguineous marriages in the area [15, 22]. The Eastern province has the highest prevalence of G6PD deficiency in Saudi Arabia in both males and females [15, 20]. The prevalence in high-risk newborns was almost similar to the earlier studies in males (26%) and in females (9.9%). There is an indication of a gradual increase in the prevalence of G6PD deficiency, with the highest recorded in the age group older than one year where the overall prevalence was (24.7%) rather than the newborn age group (18.8%), as shown in both Tables 1 and 2. This observation might also be an indication that the study was conducted within or closer to this age group. In this study, male children were much more affected than female children. This further reaffirmed the natural history of G6PD deficiency of being an X-linked recessive disorder, as well as the fact that only male hemizygotes and female homozygotes are the individuals most often affected. Heterozygous females can also be affected by the inactivation of one X chromosome through the Lyon hypothesis [23]. Notwithstanding the high prevalence of G6PD deficiency in the community, we lack the newborn screening (NBS) program to determine the true prevalence prospectively. The NBS program is invaluable, not only to determine the prevalence of G6PD deficiency but also plans for future monitoring for jaundice, to prevent acute encephalopathy from hyperbilirubinemia encephalopathy, and consequent observation for future hemolytic episodes [24]. Further family health education regarding chemicals, drugs, and food items that could adversely affect the patients leading to red blood cell hemolysis, jaundice, and anemia due to G6PD deficiency should be implemented. Quantitative measurement of the enzyme is also important to determine the World Health Organization (WHO) class of the disease to predict the natural course of G6PD deficiency [25].

## Conclusions

The present study confirms the high prevalence of G6PD deficiency in our community. We need to establish an NBS program, according to the earlier WHO recommendation to screen for G6PD deficiency if the prevalence is above 3%. Another recommendation is to screen the G6PD status during routine vaccination for those who missed NBS to enable health practitioners to protect children from preventable hemolytic episodes.
